# Removal of Polymer Clips From the Gallbladder Fossa in a Patient With Ehlers-Danlos Syndrome (EDS) to Treat Mast Cell Activation Syndrome (MCAS): A Case Report

**DOI:** 10.7759/cureus.33704

**Published:** 2023-01-12

**Authors:** Yitong Xiao, Dale A Calixte, Elizabeth Fry, Frederick Tiesenga

**Affiliations:** 1 Surgery, Saint James School of Medicine, Chicago, USA; 2 Clinical Sciences, St George’s University School of Medicine, Chicago, USA; 3 General Surgery, West Suburban Medical Center, Chicago, USA

**Keywords:** hypersensitive reaction, noninvasive surgery, foreign body removal, mast cell activation syndrome, ehlers-danlos syndrome

## Abstract

Ehlers-Danlos syndrome (EDS) is a group of hereditary disorders characterized by fragility of connective tissue. Clinical manifestations of the disorder involve the skin, joints, blood vessels, and other internal organs. We report the case of a 29-year-old female suffering from EDS and mast cell activation syndrome (MCAS). Her history includes multiple orthopedic surgeries leading to the worsening of her symptoms. This was determined to be due to medical implants placed during her multiple procedures predisposing her to severe immunological reactions. This case report emphasizes the importance of meticulous surgical intervention when managing patients with EDS.

## Introduction

Ehlers-Danlos syndrome (EDS) comprises a heterogenous group of connective tissue disorders. It is classified into six main types including classical, vascular, hypermobile, arthrochalasis, kyphoscoliotic, and dermatosparaxis with different mutations in the gene responsible for the synthesis of collagen. Clinical presentation is variable; but is not limited to skin hyperextensibility, impaired wound healing, ecchymosis and hematomas, joint hypermobility, vascular fragility, and gastrointestinal (GI) symptoms [1]. The prevalence of classical type EDS is estimated at 1/20,000, but it is likely patients with mild manifestations of the disease go undetected [2]. Systemic features of EDS include chronic pain, GI dysmotility, dysautonomia, mast cell activation, and anxiety [3]. A study conducted in 2021 by Wang et al. showed that there was a statistically significant increase of mast cell activation syndrome (MCAS) in patients diagnosed with EDS and postural orthostatic tachycardia syndrome (POTS) [4]. The association between these disorders is unclear [5]. Diagnosis of EDS is difficult due to clinical variability, lack of knowledge about this rare disorder, and vague diagnostic criteria. Nevertheless, the clinical management of EDS usually involves interdisciplinary medical specialties such as immunology, internal medicine, and surgical intervention to resolve specific symptoms of the disorder as reported in our case. 

The patient in the case presented was struggling with hypersensitivity reactions to medical implants from her extensive orthopedic surgical history related to progression of EDS. MCAS is a known complication documented in patients with EDS. After careful removal of polymer clips left over from a cholecystectomy performed five years ago, the patient reported resolution of all hypersensitivity reaction symptoms without any postoperative complications. This case study reviews the importance of noninvasive surgical intervention in a patient with EDS to avoid complications such as bleeding and spontaneous perforations [6]. 

## Case presentation

A 29-year-old Caucasian female patient presented to the surgery out-patient clinic requesting for foreign body removal. The patient was diagnosed with EDS and MCAS when she was a child. During her lifetime, the patient has suffered from endless anaphylactic reactions due to immunological hypersensitivity of MCAS. According to the patient, her clinical symptoms include nausea, vomiting, constipation, intestinal swelling, migraines, facial edema, hives, acne, fatigue, bloating, fever, chills, night sweats, and acute pulmonary inflammatory reactions. The patient stated that she has had subclinical symptoms of MCAS since childhood that became more problematic when she reached puberty. The patient’s past medical history is remarkable for EDS, MCAS, postural orthostatic hypotension, mitral valve regurgitation, tricuspid valve regurgitation, atrial fibrillation, brain fenestration (frontal lobe), pineal gland cyst, migraines, cystinuria, hypothyroidism, anemia, hypokalemia, vitamin B-12 deficiency, menorrhagia, celiac disease, osteopenia in the right hip, bowel hypomotility, post-traumatic stress disorder (PTSD), mild urinary bladder prolapse, and MCAS-induced asthma. The patient’s past surgical history includes tonsillectomy, lithotripsy, plica shelf removal of right knee, medial patella-femoral ligament (MPFL) reconstruction and lateral patella-femoral ligament (LPFL) reconstruction cadaver graft and stabilization of right knee, peroneal tendon cadaver graft and deltoid debridement of left ankle, peroneal tendon cadaver graft of right ankle, hardware removal and replacement of right ankle, labrum repair of right hip, labrum hardware removal of right hip, 360 degree labrum repair of right shoulder, hardware repair and replacement of right shoulder, 360 degree labrum repair of left shoulder, cholecystectomy, and gallbladder clip removal.

According to the patient, allergies include plastic and synthetic surgical materials, latex, plastic medical tape, soy, lecithin, gluten, wheat dextrin and wheat derived maltodextrin, seafood, mold, nickel, butter, sea salt, beef, tomatoes, watermelon, kiwis, banana, yellow #5, eggs, cheese, and phytoestrogens. She also reports hypersensitivity reactions to the flu vaccine, amoxicillin, Augmentin, Bactrim, Betadine, Bystolic, Cefaclor, Corlanor/ivabradine, duloxetine, gabapentin, iodine, ketotifen fumarate, Marcaine HCl/bupivacaine, methylprednisolone, midodrine, Motegrity, Norco, propranolol, pyridostigmine bromide, ranitidine, and tramadol. Currently, the patient’s medications include acetaminophen/butalbital, Allegra-D, Ativan, Benadryl, Bisoprolol, buffered salt tablets, calamine, cromolyn sodium, desonide cream, topical drysol, Epi-pen, Errin, fludrocortisone, glucose tabs, Linzess, loratadine, montelukast, mupirocin, naproxen, olopatadine, Pennsaid, Pepcid, Percocet, Plaquenil, potassium chloride, quercetin, Robaxin, triamcinolone cream, Ubrelvy, IM B12, Xolair injections, Xyzal, Zofran, Zyrtec, and toradol injections. Family medical history includes maternal EDS. She has no history of smoking or alcohol use. 

Due to the pathological progression of EDS, the patient underwent multiple orthopedic procedures for her hip, knee, shoulders, and ankles. After the procedures, she started having worsening symptoms such as increased joint pain and facial swelling. After looking through the patient’s past medical and surgical history, her immunologist concluded that the aggravation of symptoms was strongly associated with medical implants placed during the procedures that triggered mast cells to initiate an immune response. The patient was advised to remove the foreign bodies which were implanted. This report highlights the removal of three Weck clips that were placed during her cholecystectomy. 

**Figure 1:**
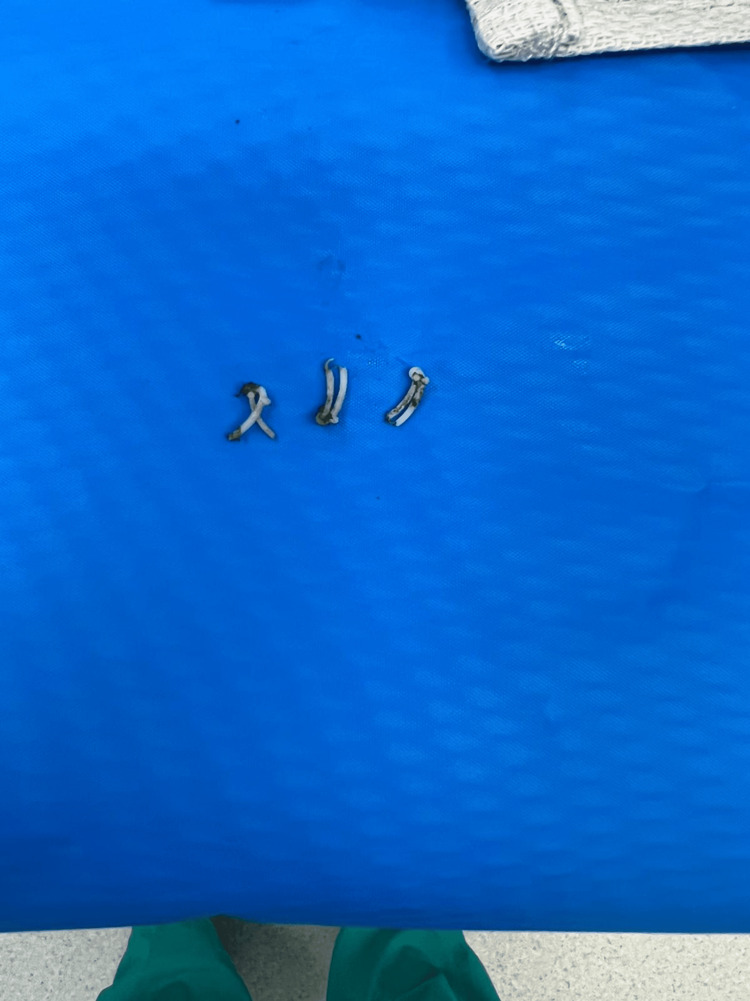
Three separate polymer Weck clips were removed from the gallbladder fossa.

On the day of the operation, she was taken to the operating room, and prepped under general anesthesia. At this time, pneumoperitoneum was obtained periumbilically, and the abdomen was explored. Epigastric, midepigastric, and right mid quadrant ports were placed percutaneously under direct vision. During the procedure, fluoroscopy was utilized and there was no evidence of metal clips in the right upper quadrant. Next, the gallbladder fossa was examined where three separate Weck clips were found. Two clips were located on the cystic duct and one on the cystic artery. All clips were removed and sent to pathology (Figure 1). No bleeding or bile spill was encountered. The patient tolerated the procedure well and was taken to the recovery room in stable condition. A few hours after the procedure, the patient was discharged without any postoperative complications. After one week, the patient returned to the out-patient clinic for a follow-up and stated that she had felt great relief of her hypersensitivity reactions since the procedure.

## Discussion

Patients with EDS display a diversified group of conditions characterized by skin hyperextensibility, atrophic scarring, joint hypermobility, and generalized tissue fragility [7-8]. There are many complications associated with EDS including hypersensitivity reactions and postural orthostatic hypotension. The knowledge of the clinical presentation of different variants associated with EDS is necessary in helping clinicians identify varying patterns of inheritance, severity, and type of collagen affected in order to provide treatment to the specific symptoms and their respective manifestations.

Mast cell activation syndrome refers to a group of disorders with diverse causes involving the skin and multiple organ systems such as the gastrointestinal, cardiovascular, respiratory, and neurologic systems. MCAs can also be classified as primary, secondary, and idiopathic. Criteria of diagnosis are based on insult on two or more organ system with urticaria, angioedema, flushing, nausea, vomiting, diarrhea, abdominal cramping, hypotensive syncope, tachycardia, wheezing, conjunctival injection, pruritus, and nasal stuffiness [9]. The clinical features of MCAS can be presented when mast cells are abnormally regulated in the setting of mast cell disorders or affect induced by neoplastic gain-of-function mutation, most commonly in KIT, a transmembrane receptor tyrosine kinase highly expressed by mast cells. The latter can impede normal tissue function and release mediators incongruously, resulting in a series of localized and systemic symptoms. Clinical features of MCAS can also be predominant when mast cells are activated disproportionately to mitigate insults from infections, physical triggers, venoms, or foreign bodies [10].

Treatment modalities for MCAS mainly revolves around supportive care. Symptoms of MCAS can be managed by blockade of mediator receptors (H1 and H2 antihistamines, leukotriene receptor blockade), inhibition of mediator synthesis (aspirin, zileuton), mediator release (sodium cromolyn), and anti-IgE therapy [10]. Other management includes avoidance of triggers such as food, medications, allergens, and inhalational triggers of mast cell activation exposure to allergens. Recent clinical trial investigated the use of signal transduction inhibitors, imatinib, which resulted in a decrease in mast cell tryptase levels and mast cell numbers [11].

Other studies have well documented the association between EDS and MCAS; according to a studies conducted by Wang et al., a total of 195 medical records of demographics of the patients and diagnoses of POTS, EDS, or MCAS were recorded. The percentage of MCAS within the group of POTS and EDS was 31% in comparison with 2% within the non‐POTS and EDS group. The 95% confidence interval calculated for the MCAS in the POTS and EDS group did not overlap with 2%, which showed a statistically significant result. There was a marked percentage of MCAS among the patients with diagnoses of POTS and EDS [12].

Among the most common complications of surgical treatment associated in patients with EDS are vessel ruptures and spontaneous perforations. Therefore, careful preoperative indications should be considered when evaluating patients with EDS for surgical treatment. Moreover, clinicians need to be aware of patients’ past medical history and prior history of MCAS. Often, a nonsurgical approach can be the best treatment approach for these patients, depending on the condition [13].

Previous studies have documented the use of the Weck polymer clips during laparoscopic intervention and their efficacy over the use of metal automatic clip appliers [4, 14]. The Weck polymer clips consist of a permanent, non-absorbable, non-conductive system that enhances the secure ligation of vessels up to 10 mm and permits surgeons to keep the procedure as minimally invasive as possible in a laparoscopic cholecystectomy [15]. Our patient’s history of ongoing anaphylactic reactions due to immunological hypersensitivity of MCAS is a known complication of EDS and warrants further investigation into the clinical picture and pathology related to her presentation. We hypothesized that the Weck polymer clips are associated with the immunologic reactions the patient presented with. Moreover, the underlying collagen abnormalities related to the patient’s condition greatly put the patient at high risk of poor cutaneous wound healing and dehiscence [16]. 

## Conclusions

Ehlers-Danlos syndrome is a diversified group of connective tissue disorders with varying manifestations including hypermobility EDS and vascular EDS, which are characterized by defective collagen synthesis and processing. Symptoms include different degrees of hyperextensive skin, joint hypermobility, and tissue fragility. MCAS refers to a group of disorders with diverse causes presenting with symptoms involving multiple organ systems and the skin as the result of mast cell mediator release. We reported a rare case of hypersensitivity to permanent polymer clips placed during a cholecystectomy in a patient with EDS with a turbulent medical history of MCAS. This case report provides valuable insight to clinicians in the management of patients with EDS and history of MCAS with prior surgical intervention using polymer clips. Despite introduction of diagnostic criteria and some advances in treatment in the last decade, further investigation is needed to provide more insight with the medical and surgical management of patients with connective tissue disorders and MCAS.
